# E-Learning Is Not Inferior to On-Site Teaching in a Psychiatric Examination Course

**DOI:** 10.3389/fpsyt.2021.624005

**Published:** 2021-04-13

**Authors:** Christoph Rauch, Janine Utz, Miriam Rauch, Johannes Kornhuber, Philipp Spitzer

**Affiliations:** ^1^Department of Psychiatry and Psychotherapy, Friedrich-Alexander-University of Erlangen-Nuremberg, Erlangen, Germany; ^2^Department for Vascular Surgery, Neumarkt Hospital, Neumarkt in der Oberpfalz, Germany

**Keywords:** digital teaching, online learning, medical education, simulated patients, communication skills, psychiatry, on-site teaching, practical course

## Abstract

**Background:** Implementing e-learning into medical education is a growing field of research. Researchers have had positive experiences so far, and evidence suggests it to be no less effective than offline teaching. However, there are a few findings concerning psychiatric education and the use of simulated patients online.

**Methods:** We developed an online workshop for medical students at our psychiatric clinic, including group work exercises, lectures, and interviews with simulated patients. To compare the learning outcome, a cohort of students learning online was compared with a previous cohort that learned on-site. The same objective structured clinical examination (OSCE) was used in both cases. Evaluation questionnaires were gathered from students and lecturers and were compared with the former semesters along with the exam results.

**Results:** The exam grades did not significantly differ between on-site and online teaching, even though students rated their own communication skills better with online teaching. We also found that the connection experienced between students and teachers was impaired without on-site contact.

**Discussion:** We conclude that an online course may be an effective alternative to on-site teaching but requires further improvement to maintain a dependable student–teacher relationship.

## Introduction

For many years, e-learning has been a growing field. In medical education, patient interaction and the special skill set of physicians call for specific teaching methods. There are several reports showing that e-learning is as effective as conventional lectures and that even practical courses can successfully be held online. However, evidence is still lacking (1–3).

Specifically, there are a few findings regarding the teaching of psychiatric examination and communication skills online. When considering the constructive alignment of the learning process, it becomes clear that communication can only be learned by communicating (4). While the use of simulated patients to teach and test practical and communicative skills is well-established (5–7), it is unclear whether it can be transferred to an online learning environment with video conferences.

When discussing digital teaching alternatives, the students' as well as the lecturers' preferences should be considered (8, 9).

We transferred the contents of our practical course program, including the exam, to video conferences and our university's digital platform. Students were asked to answer a questionnaire about the course afterward.

Purpose of the present study:

In this study,

- we explored whether it is possible to create an online course that teaches psychiatric history taking, examination, and communication to fourth year medical students.
- we compared whether the exam results of students who were taught exclusively online differ from those taught in our established on-site course.
- we investigated whether the teaching modalities had an impact on student satisfaction.
- we assessed how the online learning environment is accepted by both lecturers and students.

## Methods

### Participants

Fourth-year medical students complete an obligatory 1-week practical course at our psychiatric clinic. In the winter semester of 2019/20, when the course was held on-site, we examined 156 students in an objective structured clinical examination (OSCE) of which 150 (return rate: 96% female: 87, male: 54, unknown: 9) participated in the questionnaire. For the online teaching summer semester of 2020, 175 students were examined and 156 (return rate: 89% female: 93, male: 65, unknown: 2) participated in the questionnaire.

Additionally, we questioned all 16 (return rate: 75%) lecturers teaching in the summer semester about their experiences. The lecturers were the same in the on-site and online semesters, with the exception of four lecturers due to clinical rotations.

The participating medical students and lecturers voluntarily agreed to participate in the survey. The students were explicitly informed that participation would not impact their exam grades and that the questionnaire answers and grades would be processed anonymously. This study was submitted to the ethical committee of the Friedrich-Alexander University Erlangen-Nuremberg and received a designation of exempt.

### Course Concept

The practical education consists of 5 course days and the following OSCE. Each day includes a 45-min theoretical seminar about common psychiatric syndromes presented by one of our senior physicians. In previous semesters, two to three real and/or simulated patients were then interviewed by the students in groups of seven on the wards. During the online semester, we used conference calls, in which only simulated patients were available due to privacy protection concerns. The actors, lecturers, and students gave feedback based on their perception of the interview. We expanded the course in the digital setting by offering a 20-min group exercise every day. Additionally, we offered a quiz for self-testing and attached resources based on our online lecture content such as podcasts and reading material (5). After 1 week, one of four standardized patient cases was given to the students as an OSCE. In the summer semester of 2020, this OSCE was also carried out via video conference.

### Simulated Patients

The pool of simulated patients includes 6 professional actors and 10 amateurs, with a mean of seven semesters of experience as a simulated patient (5 males, 11 females) and around 16 assignments in each semester. Roles played include dementia, delirium, schizophrenia, major depression, bipolar disorder, adaptive disorder, obsessive–compulsive disorder, anxiety disorder, sexual arousal disorder, and sexual dysfunction. They are regularly trained and supervised in their roles, as well as in giving feedback to the students.

### Objective Structured Clinical Examination Testing

Simulated patients will perform one out of four clinical cases (dementia, schizophrenia, bipolar disorder (current depressive episode), obsessive–compulsive disorder). The examiners are not the same as the lecturers. To provide a fair exam, we use a standardized questionnaire, rating performance of communication models, medical thoroughness, and the following case presentation (Supplementary Figure 1). The time frame of the interview was 15 min plus 90-s patient presentation. We taught and examined two communication models: the WRMS model (Wait-Repeat-Mirror-Summarize), used to support active listening (10), and the more advanced NURSE model (Naming-Understanding-Respecting-Supporting-Exploring Emotions), aiding the use of emotions in communication (11). One of the four cases (major depression/bipolar disorder) included taking the sexual history of patients. The scores we compared can be seen in Table 1. The same OSCE has been performed in the last four semesters, showing no improvement of the overall results over time. This indicates that previous knowledge of the exam structure is not advantageous to the students' performance; rather, their actual subject knowledge is examined.

**Table 1:** OSCE performance scores: composition of the compared scores with exemplary tested items and achievable number of points.

**Score**	**Exemplary items**	**Achievable points**	**Comment**
Total score		60	Interview and patient presentation
WRMS	Applies the WRMS model	4	Wait-Repeat-Mirror-Summarize
NURSE	Applies the NURSE model	5	Naming-Understanding-Respecting-Supporting-Exploring Emotions
Psychopathological symptoms	Assesses orientation; asks for delusions; examines perception	16 (12 for the bipolar disorder case)	Included only the most relevant items for the case, not a complete assessment
Sexual history	Asks for sexual arousal disorders adequately; asks for sexual dysfunction adequately	4	Only assessed in the bipolar disorder case OSCE
Patient presentation	Describes current complaints proposes correct treatment attempt; keeps 90-s time limit	20	

### Questionnaire

The students answered the questionnaire following completion of the course and prior to the exam. We assessed 15 items, including interest, motivation, self-assessment, in-course feedback and overall enjoyment. The rating was based on German school grades [1 (totally agree) to 6 (totally disagree)]. The students' gender and individual study duration were collected as demographic data (Supplementary Figure 2).

Furthermore, students were asked to compare the online course with their previous on-site learning experience (better–same–worse). Lecturers were asked to compare the online course to the previous semesters (better–same–worse).

### Digital Environment

We used Zoom (Zoom Version 5.0.2., Zoom Video Communications Inc., California, USA) as a platform for conducting video conferences.

Ilias StudOn (ILIAS Version 5.4.17, ILIAS open-source e-Learning e.V., Cologne, Germany) is the online e-learning system of the University of Erlangen-Nuremberg.

### Data Analysis

Statistical analysis was performed using SPSS (SPSS 21, IBM, Armonk USA). We used hierarchic linear modeling (HLM 7, Scientific Software International, Inc., Skokie, USA). Level-1 variables describe data collected from a single student (gender, duration of study), while level-2 variables consider data collected in the same week (lecturer, simulated patients, examiner, OSCE case, online/on-site teaching) (12). Results of the hierarchic linear modeling are reported as the difference between slopes (b) of the regression curves and its significance (p).

## Results

### The Test Results Did Not Differ Between Online and On-Site Learning

We compared the total number of points achieved in the OSCE as well as various sub-scores in the last on-site semester (winter semester 2019/20, *n* = 150) with those achieved in the e-learning semester (summer semester 2020, *n* = 156). The result of the power calculation indicated that with a total *n* of 306, small-to-medium effect sizes could be detected (ρ = 0.185). The intercept-only model revealed an ICC of 0.385. Thus, 38.5% of the variance in total exam score is between groups (including variables such as the semester, the lecturer, and the examiner) and 61.5% of the variance in the total exam score is between students (including variables such as age and duration of study) within a given group.

**Figure 1:**
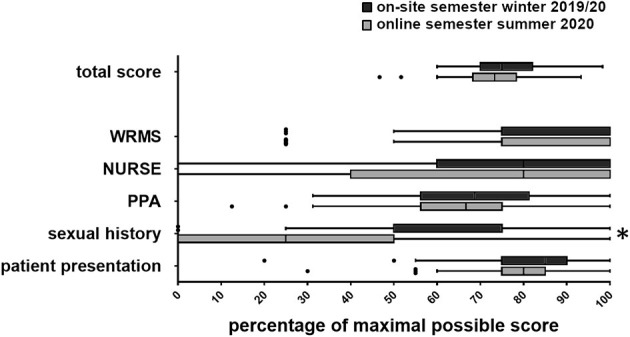
Tukey plot of the OSCE results with sub scores (WRMS, Wait-Repeat-Mirror-Summarize-Model; NURSE, Naming-Understanding-Respecting-Supporting-Exploring-Emotions-Model; PPA, Psychopathological Assessment; Sexual history; Patient presentation). *p < 0.05.

There was no significant difference in the total score (*b* = −1.47, *p* = 0.127), the communication scores (WRMS: *b* = −0.12, *p* = 0.425; NURSE: *b* < −0.01, *p* = 0.991), the psychopathological symptoms score (*b* = −0.28, *p* = 0.561), or the clinical presentation (*b* = −0.39, *p* = 0.306). However, there was a significant difference in the taking of the patient's sexual history (*b* = −0.91, *p* = 0.011), meaning that the students of the on-site semester performed better at this task than the students of the e-learning semester (Figure 1).

### Gender Differences in the Objective Structured Clinical Examination Score

The students' gender significantly affected the exam results when including data from both semesters (*b* = 1.57, *p* = 0.003). In the subsequent analysis of the individual semesters, we discovered no significant relationship between gender and total score in the on-site semester (*b* = 0.73, *p* = 0.290), while there was a significant relationship between gender and total score in the online semester (*b* = 2.43, *p* = 0.002). Male students scored significantly fewer points in the online semester. We analyzed the relationship between gender and total score with consideration of the teaching method (online vs. on-site) as a potential moderator/mediator. In the analysis, we observed a difference between the estimation of fixed effects and the estimation of fixed effects with robust standard errors. Therefore, we decided to use the more conservative approach and report the results of the fixed effects with robust standard errors. There was a significant relationship between the method (online vs. on-site) and the total score (*b* = −4.42, *p* = 0.023), as well as a significant cross-level interaction between method and gender (*b* = 1.90, *p* = 0.042), whereas the relationship between gender and total score was no longer significant (*b* = 0.49, *p* = 0.495). This suggests that the method (online vs. on-site) is a mediator in the relationship between gender and total score: there is no stand-alone effect of gender on the total score; the effect is completely mediated by the fact that male students had worse total scores only in the online course environment.

### On-Site Learning Receives Better Rating in Student–Teacher Relationship Items and Structure

The return rate of the student questionnaire was 98% for the on-site winter semester (*n* = 153) and 89% for the online summer semester (*n* = 156).

**Figure 2:**
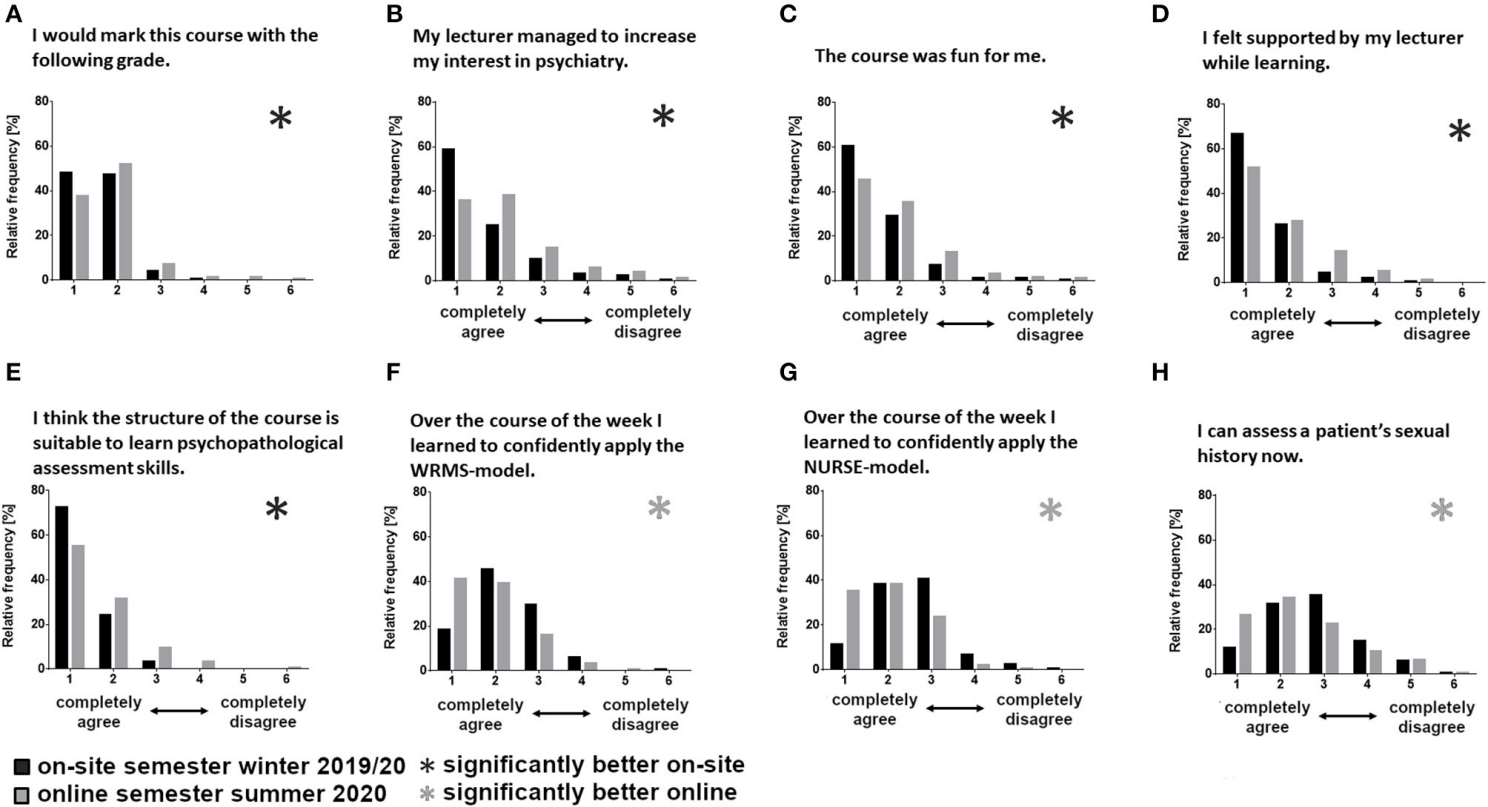
Distribution of questionnaire ratings sorted by item **(A–H)**, comparing between the semesters (winter semester 2019/20, *n* = 150; summer semester 2020, *n* = 156) using hierarchic linear modeling (*significant differences between the semesters; WRMS, Wait-Repeat-Mirror-Summarize-Model; NURSE, Naming-Understanding-Respecting-Supporting-Exploring-Emotions-Model).

During the on-site semester, the students rated the following items better: the subjective support they received from their lecturer (*b* = 0.37, *p* = 0.017, Figure 2D), how much their interest in psychiatry was increased by the lecturer (*b* = 0.39, *p* = 0.030, Figure 2B), how much they enjoyed the course (*b* = 0.28, *p* = 0.031, Figure 2C), as well as how they would grade the course overall (*b* = 0.20, *p* = 0.034, Figure 2A). However, as previously reported, these differences did not impact the exam grade.

### With E-Learning, Students' Self-Assessment in Communication Is Higher

Contrarily, students' self-assessment in the online semester improved for three communication heavy skills: the use of the WRMS strategy (*b* = −0.43, *p* = 0.002, Figure 2F), the use of the NURSE strategy (*b* = −0.61, *p* < 0.001, Figure 2G), and their ability to take the sexual history of a patient (*b* = −0.40, *p* = 0.035, Figure 2H).

### Acceptance and Preference

The questionnaire item “I think the structure of the course is suited to teaching psychiatric expertise” was rated better for the on-site semester (*b* = 0.32, *p* = 0.003, Figure 2E).

When asked to compare the online course with their previous on-site learning experience, 50.0% of the students of the summer semester preferred the on-site semester, 24.2% preferred online teaching, and 25.8% rated them equally (*n* = 120).

Lecturer feedback had a return rate of 75%. Seventy-five percent of the lecturers preferred the on-site semester, 8% preferred the online semester, and 17% rated them equally (*n* = 12).

## Discussion

Overall, we did not observe a difference in the exam scores between the on-site and the online semesters, except for the taking of the sexual history. The questionnaire showed that while students rated their own communication skills better in the online semester, they felt less connected to their lecturer.

### It Is Possible to Create an Exclusively Digital Course

The existing digital structures were flexible enough in executing our plans to combine video conferences with online group work, as well as providing online materials for self-study. Zoom proved viable in imitating a telemedical setting. The online learning tool of the university, based on ILIAS, provided options to implement resources and assignments in a variety of media formats (weblinks, pictures, videos, podcasts, and texts), allowing for a more diverse learning experience. Students, lecturers, and actors had no issues using the platforms after an introduction. Technical problems did not occur on a relevant scale. Thus, the realization of the project did not meet many obstacles. As a result, the exam and questionnaire results were interesting to observe.

### The Only Difference in the Test Results Was the Taking of the Sexual History When Comparing On-Site With Online Learning

The students' overall performance and the sub-scores were not relevantly impaired in the exclusive online teaching environment.

The only score that significantly changed was the taking of the sexual history. It is worth noting that this score is tested only in one-fourth of the exams (major depression/bipolar disorder). Wide fluctuations of this score have therefore also been observed in previous semesters. However, since sexual history often is a difficult topic to address (13), one could argue that the training lacked in thoroughness, the lecturers expressed more personal reservations in the online setting, or the online setting was indeed unsuitable in forming a dependable student–teacher relationship. Regarding the latter, Palmer et al. (14) created an online course to train students to interview patients about adverse sexual effects of SSRIs and observed positive communicative developments. This could indicate that the online setting itself is not at fault, rather, the degree to which our lecturers adapted the teaching of the sexual history taking to an online environment. We hypothesize that reservations (e.g., shame) regarding talking about sexuality may be harder to overcome in an online environment, and this impacted the quality of the teaching.

Overall, the results did not indicate a difference in the general student performance, thus, suggesting that the transfer of our course to an online setting did not have a considerable impact on the learning effectiveness.

Several authors studying the quality and practicability of online medical teaching reported similar findings. There is a wide range of methods described by the term “web-based learning,” complicating the navigation of literature (15). Our study included multiple such approaches, which were mostly tested as a stand-alone intervention. There are several findings that video lectures and live lectures are equally effective in teaching new content (1, 16, 17). Providing digital content, in addition to lectures and simulated patient interviews, is a useful tool to promote deep learning (18, 19). Topic-focused interventions can improve specific skills, such as taking the patient's sexual history (20, 21). The core of our course is the training of communication skills. Kaltman et al. (22) found that students trained with interactive online videos demonstrated more of the taught patient-centered interview skills in a face-to-face. The usefulness of simulations in medical education has been long since established (23). Parisi et al. used an approach using Zoom in small groups with simulated patients with diabetes, similarly to our study. They found it to be effective as well, but emphasized the lack of non-verbal communication training, which is especially important in intercultural contexts (24). Mohos et al. found the transfer of their course to an online environment to be a success, with consistent outcomes compared with previous semesters. Nevertheless, they raised concerns considering the missing personal contact (25), which we came across as well.

### The Subjective Learning Experience Seems to Differ Between Online and On-Site Teaching

The students evaluated the on-site semester better in student–teacher-related items (subjective support from the teacher, increased interest in psychiatry, and overall enjoyment), as well as the course as a whole. This may indicate that the establishment of face-to-face contact with the lecturer plays a significant role in the perception of the experience as pleasant and helpful (26). As the participants' satisfaction influences the success of a didactic method (on-site/online), it may be considered that while the exam results stayed the same, the course did not entirely meet its standards (27). Thus, it should be aimed to include elements in online courses in which space for such interaction is provided (28). For example, an introductory round at the start of the week, as well as daily flash feedback rounds could be included to promote group synergy. More time for discussion of the topic could further improve this. With respect to the structure of the course, we repeatedly received verbal feedback emphasizing that it is preferable if the lecturer does not switch during the week.

In the online semester, the students self-assessed their communication skills (WRMS, NURSE, and SEX) better. It is notable that only the communication strategies but no other learning content, such as the examination of symptoms or the clinical presentation, were rated differently. This may have various reasons. The simulated patients may have been primed to react to these communication models in a more obvious way. It is also possible that without proper non-verbal communication, the students had to rely on the learned strategies or could better focus on the patient's reaction. Perhaps the lowered social anxiety allowed for bolder self-assessment. Special consideration should be given to the self-assessment of summer students, who rated their sexual history taking skills higher than the previous semester but achieved lower scores in their exams. This may indicate that the self-assessment of students, at least in the form tested in this study, is not reliable when compared with the results of an OSCE (29).

### Differences Regarding Gender

The method (online vs. on-site) is a mediator in the relationship between gender and total score. We found that female students reached a significantly higher average total score in the online semester. We cannot conclude whether this is because female students performed better during the online semester or if male students struggled more, neither whether it was directly caused by our e-learning method or surrounding circumstances. González-Gómez et al. (30) found that female students were more accepting of e-learning, which might be the reason for their better performance in the OSCE during the online semester. Thus, gender differences should be considered when investigating e-learning in educational research, as findings similar to ours have been reported before (31, 32).

### Students' and Lecturers' Reservations Toward E-Learning Are to Be Considered

We also observed that the majority of students (50%) and lecturers (75%) of the summer semester preferred on-site teaching. Students also thought that the structure of the on-site course is more suited to teach psychiatric expertise.

Mullins et al. (33) also found that students preferred on-site psychiatric lectures, even though they liked the flexibility and availability of online lectures. While there is still a preference for traditional teaching methods, the acceptance of online teaching methods is increasing, and participants adapt well when confronted with them (34, 35). Nevertheless, the lecturers' willingness and trust in the teaching method has a relevant role in its proper implementation (9).

### Limitations and Further Research

The power analysis showed that we had a sufficient number of participants, but it should be considered that the summer semester of 2020, at the time of our intervention, was also severely influenced by the COVID-19 pandemic, which may have had a number of unforeseen effects on our results (36–39). Also, our groups where not randomized or controlled (40).

The OSCE testing we performed has been validated throughout the previous four semesters and has proved reliable. However, it cannot be excluded that conducting the exam online has an effect on the examiner that mingles with the influences of our intervention.

A general problem is that the variance of the lecturer, student, and examiner has a significant impact on the outcome of learning and exam results. The influence of the teaching method we investigated may be overshadowed by these variables.

## Conclusion

Overall, we can conclude that a practical course in psychiatry can be conducted in an online setting with video conferences. The exam grades did not differ in comparison to on-site teaching. Items illustrating the student–teacher relationship were rated better when teaching in person, indicating that this is an issue to be considered in future studies.

## Data Availability Statement

The raw data supporting the conclusions of this article will be made available by the authors, without undue reservation.

## Ethics Statement

The participating medical students and lecturers voluntarily agreed to participate in the survey. The participation did not impact the exam grades, and the questionnaire answers and grade were processed anonymously. This study was submitted to the ethical committee of the Friedrich Alexander Universität Erlangen-Nuremberg and received a designation of exempt.

## Author Contributions

CR, JU, MR, JK, and PS designed the study. CR, JU, and PS collected the data. Data were analyzed and evaluated by CR, JU, MR, JK, and PS. The statistics were carried out by CR and MR. CR, JU, MR, and PS drafted the manuscript. All authors critically reviewed the manuscript and provided constructive comments to improve the quality of the manuscript. All authors have read and approved the final manuscript. All authors agree to be accountable for all aspects of the work.

## Conflict of Interest

The authors declare that the research was conducted in the absence of any commercial or financial relationships that could be construed as a potential conflict of interest.
