# Supplementary figures and images for: E-Learning Is Not Inferior to On-Site Teaching in a Psychiatric Examination Course
Source: Front Psychiatry. 2021 Apr 13;12:624005. doi: 10.3389/fpsyt.2021.624005 (PMC8076569; doi:10.3389/fpsyt.2021.624005)

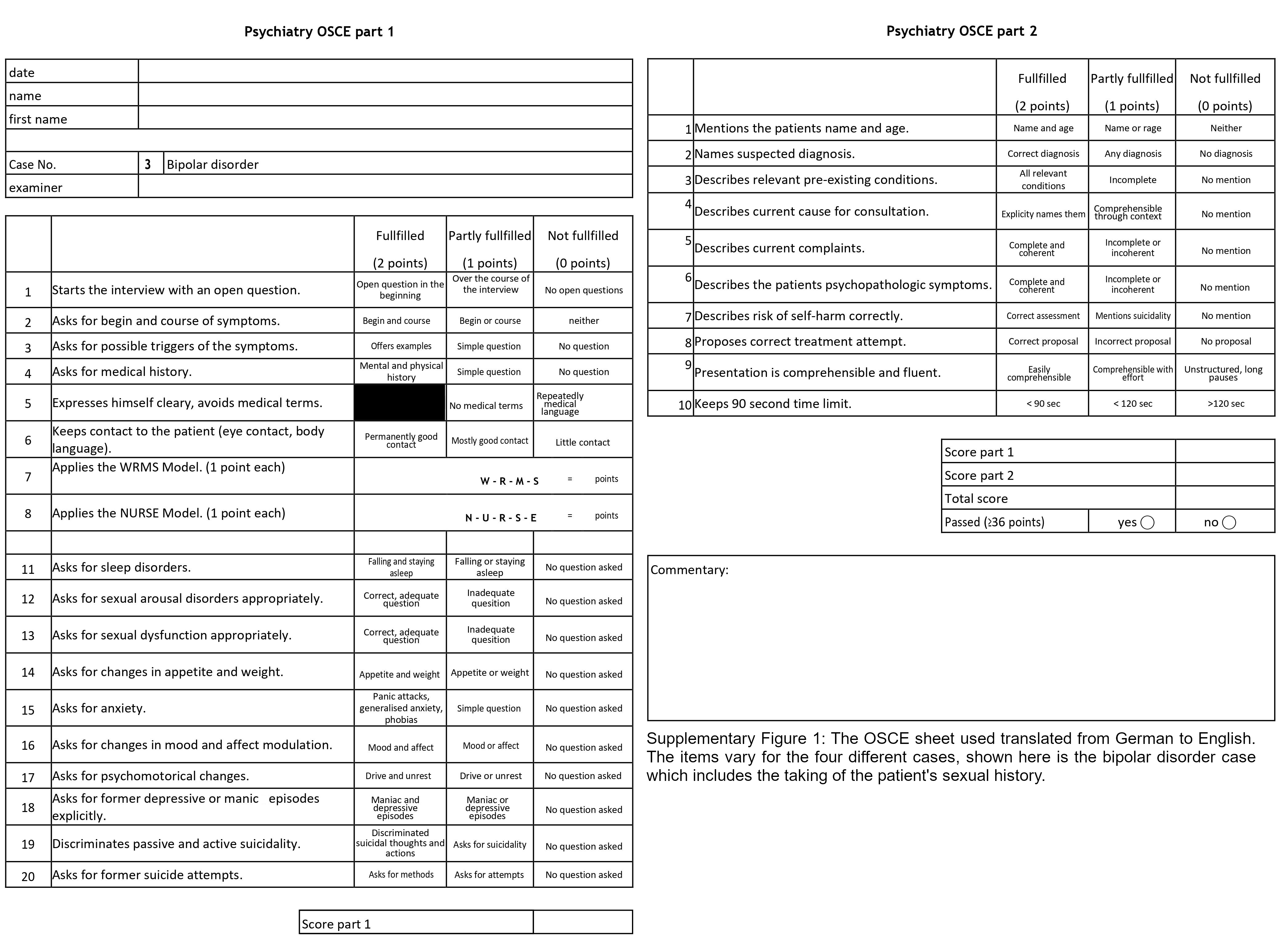

Supplement: Supplementary file 1 [file Image_1.TIFF]

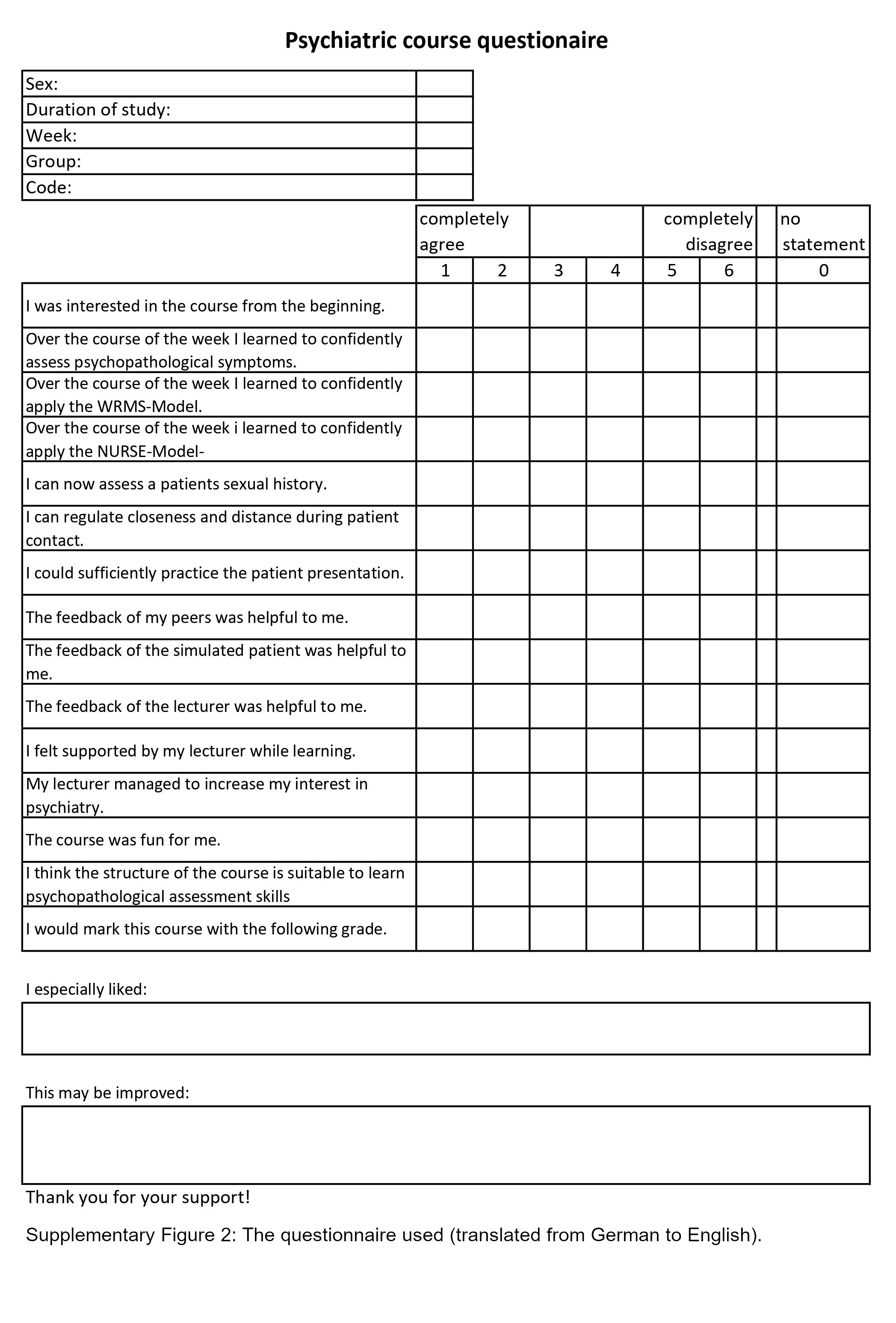

Supplement: Supplementary file 2 [file Image_2.TIFF]
